# Acoustic Radiation Force Impulse Imaging for Non-Invasive Assessment of Renal Histopathology in Chronic Kidney Disease

**DOI:** 10.1371/journal.pone.0115051

**Published:** 2014-12-29

**Authors:** Qiao Hu, Xiao-Yan Wang, Hong-Guang He, Hai-Ming Wei, Li-Ke Kang, Gui-Can Qin

**Affiliations:** 1 Department of Diagnostic Ultrasound, the People's Hospital of Guangxi Zhuang Autonomous Region, Nanning, China; 2 Department of Nephrology, the People's Hospital of Guangxi Zhuang Autonomous Region, Nanning, China; 3 Department of Pathology, the People's Hospital of Guangxi Zhuang Autonomous Region, Nanning, China; Yonsei University College of Medicine, Republic Of Korea

## Abstract

**Objective:**

To investigate the stiffness values obtained by acoustic radiation force impulse (ARFI) quantification in assessing renal histological fibrosis of chronic kidney disease (CKD).

**Methods:**

163 patients with CKD and 32 healthy volunteers were enrolled between June 2013 and April 2014. ARFI quantification, given as shear wave velocity (SWV), was performed to measure renal parenchyma stiffness. Diagnostic performance of ARFI imaging and conventional ultrasound (US) were compared with histologic scores at renal biopsy. Intra- and inter-observer reliability of SWV measurement was analyzed.

**Results:**

In CKD patients, SWV measurements correlated significantly with pathological parameters (r = −0.422–−0.511, P<0.001), serum creatinine (r = −0.503, P<0.001), and glomerular filtration rate (r = 0.587, P<0.001). The mean SWV in kidneys with severely impaired (histologic score: ≥19 points) was significant lower than that mildly impaired (histologic score: ≤9 points), moderately impaired (histologic score: 10–18 points), and control groups (all P<0.001). Receiver operating characteristic (ROC) curves analyses indicated that the area under the ROC curve for the diagnosis of renal histological fibrosis using ARFI imaging was superior to these conventional US parameters. Using the optimal cut-off value of 2.65 m/s for the diagnosis of mildly impaired kidneys, 2.50 m/s for moderately impaired kidneys, and 2.33 m/s for severely impaired kidneys, the corresponding area under the ROC curves were 0.735, 0.744, and 0.895, respectively. Intra- and intre-observer agreement of SWV measurements were 0.709 (95% CI: 0.390–0.859, P<0.001) and 0.627 (95% CI: 0.233–0.818, P = 0.004), respectively.

**Conclusions:**

ARFI may be an effective tool for evaluating renal histological fibrosis in CKD patients.

## Introduction

Chronic kidney disease (CKD) is a major public health problem worldwide. Patients with CKD are at substantially increased risk for progressing to the end stage renal disease, cardiovascular disease, and premature death [1]–[3]. Renal biopsy remains the reference standard for identifying renal histological fibrosis and guiding therapy in patients with CKD. However, it is an invasive process associated with several complications such as silent hematoma, macroscopic hematuria, arteriovenous fistula, infection, or even death. Moreover, susceptible to sampling errors and considerable intra-observer and inter-observer variabilities produce negative effect on the accuracy of renal biopsy [4], [5].

Ultrasound (US) is one of the most frequently used techniques to evaluate renal structure and status in the diagnosis and follow-up of CKD. Renal length, parenchymal thickness, and resistive index (RI) have been reported to correlate statistically with glomerular sclerosis and tubular atrophy [6], [7]. But these parameters still lack of sensitivity and specificity in the evaluation of renal failure. Early stage of CKD is always not reflected in specific changes in the renal morphology.

Acoustic radiation force impulse (ARFI) is a novel technique of elastography that integrates in a conventional ultrasound machine and utilizes sound waves to evaluate the tissue elasticity quantitatively by measuring the shear wave velocity (SWV). It has been proposed as an alternative method for assessing liver fibrosis, thyroid nodules, breast lesions, as well as characterizing of atherosclerotic plaques and monitoring the results of radio-frequency ablation [8]–[12]. Recently, the feasibility of ARFI for evaluating the renal parenchyma elasticity was shown. Stock et al. [13] suggested that SWV is potentially independent variable for evaluating the degree of fibrosis in renal transplant. Guo et al. [14] reported that SWV was significantly correlated to estimated glomerular filtration rate (e-GFR), urea nitrogen, and serum creatinine. Whereas, in contradiction to these findings, Syversveen et al [15] stated that SWV values did not differ between kidney grafts with various degrees of fibrosis. In a study by Wang et al. [16], SWV measurements did not correlate with any pathological indicators of fibrosis and could not predict the different stages of CKD. Further studies regarding the effectiveness of ARFI elastography in detecting renal parenchyma stiffness would therefore be of interest. The purpose of our study was to compare, in a pilot study, the ARFI quantification with conventional US for the noninvasive assessment the renal histological fibrosis of CKD.

## Materials and Methods

### Study population

The study protocol was approved by the Institutional Review Board of the People's Hospital of Guangxi Zhuang Autonomous Region and written informed consent was obtained from all participants. Between June 2013 and April 2014, 163 consecutive patients with CKD (91 men, 72 women; mean age 41.3 years, age range 18–79 years) who underwent renal biopsy were enrolled. According to the guideline established by the Kidney Disease Outcomes Quality Initiative (K/DOQI) of the National Kidney Foundation (NKF), CKD was defined as either kidney damage or e-GFR below 60 ml/min/1.73 m^2^ for at least 3 months, irrespective of the cause [17]. e-GFR was calculated by serum creatinine based on the Modification of Diet in Renal Disease Study (MDRD) equation: e-GFR (ml/min/1.73 m^2^)  = 186× serum creatinine (mg/dl)^−1.154^× age (year)^−0.203^×0.742 (if female) ×1.212 (if African American) [18], [19]. Patients warrant biopsies were based on the following indications: haematuria, high or increasing level of proteinuria, elevated blood pressure, nephritic syndrome, or impaired kidney function. None of these patients had any other kidney lesions, such as stones, hydronephrosis, cysts, congenital variation, or renal tumors.

In addition, 32 healthy adult volunteers (17 women and 15 men; mean age 41.5 years, age range, 21–76 years,) who did not have any clinical signs of renal disease were invited to participate as normal control. No volunteers had abnormal laboratory tests (serum creatinine, urea nitrogen, uric acid, and urinary albumin), or had abnormal imaging findings by conventional sonography.

### Imaging acquisition

Conventional US and ARFI examinations were performed using an Acuson S2000 ultrasound machine (Siemens Medical Solutions, Mountain View, CA, USA) equipped with ARFI function (Virtual TouchTissue Quantification package). A curved array transducer (4C1, frequency range: 1–4 MHz) was applied. All measurements were performed by the same observer (Q.H., with 8 years of experience in sonographic examination), who was blinded to the clinical and pathological data.

Subjects were examined in the left lateral decubitus position. Before ARFI imaging, the length, parenchymal thickness and interlobar arterial RI of the right kidney were measured by conventional US examination. Renal length was determined as the maximum longitudinal dimension in coronal section. Parenchymal thickness was measured as the distance from the renal sinus fat to the renal capsule. Resistive index of interlobar arterial was recorded by Doppler ultrasonography. Color gain and the pulse repetition frequency were adjusted individually to avoid aliasing. The Doppler angle was set below 60° with respect to the long axis of the interlobar artery. Data are presented as means of six readings.

When performing ARFI, the probe was placed at the middle third of the renal parenchyma and the applied transducer pressure was minimized as much as possible. Once the location of target area had been determined after optimizing the B-mode image, the patient was asked to hold their breath for a moment and the Virtual Touch quantitative function was initiated. Region of interest (ROI), with fixed sizes of 1.0 cm×0.6 cm, was positioned in the outer renal cortex and carefully excluded from the renal medulla and sinus. Then, we observed the velocity and measurement depth of shear wave on the screen. The SWV measurements were expressed in meters per second. A mean value of 10 valid measurements was obtained in each subject for analyses. In the event of a non-valid measurement (displayed as X.XX m/s), a repeated measurement was carried out.

### Intra- and inter-observer reliability studies

The 32 healthy volunteers were also participated in the intra- and inter-observer reliability studies of SWV measurements. ARFI images were performed by the first operator (Q.H.) and then repeated by a second observer (G.C.Q., with 4 years of experience in sonographic examination) on the same day for assessment of inter-observer reliability. The operators were blinded to each other's examination results. Each volunteer was examined repeatedly by the first operator with 7 days interval to determine intra-observer reliability.

### Histological evaluation

Ultrasound guided 18-G Tru-Cut biopsies were conducted on the right inferior pole of the kidney parenchyma in CKD patients within 3 days after ARFI examinations. Biopsy specimens were fixed in 4% formalin and embedded in paraffin. 3-µm-thick serial sections were stained with hematoxlin-eosin, periodic acid-Schiff, Masson's trichrome, and Jones' methenamine silver stain. Each specimen contained at least 7 glomeruli. Histological analysis was assessed by two experienced pathologists (3 and 5 years of experience in nephropathology, respectively) who were blinded to the clinical data and the ARFI measurements. Any discrepancy was resolved by consensus. Biopsies were scored based on the pathological findings with glomerular sclerosis, tubulointerstitial damage, and vascular sclerosis, which have been previously introduced by Li et al. and Katafuchi et al. [5], [20] (Table 1).

**Table 1:** Index of renal histological scores.

Score	Glomerular score (3–12 points)			Tubulointerstitial score (3–9 points)			Vascular score (2–6 points)	
	Glomerular hypercellularity	Glomerular segmental lesions	Glomerular sclerosis	Interstitial cell infiltration	Interstitial fibrosis	Tubular atrophy	Vessel wall thickening	Arterial hyaline change
1	<25%	<10%	<10%	<25%	<25%	<25%	<10%	<10%
2	25%–50%	10%–25%	10%–25%	25%–50%	25%–50%	25%–50%	10%–25%	10%–25%
3	>50%–75%	>25%–50%	>25%–50%	>50%	>50%	>50%	>25%	>25%
4	>75%	>50%	>50%	NA	NA	NA	NA	NA

NA  =  not applicable.

CKD patients were then classified according to the histologic scores: mildly impaired (≤9 points); moderately impaired (10–18 points); severely impaired (≥19 points).

### Statistical analysis

SPSS version 13.0 statistical software package (SPSS, Chicago, IL, USA) was used for data analysis. The mean SWVs, renal length, parenchymal thickness, and interlobar arterial RI among different groups were analyzed by one-way analysis of variance (ANOVA) and the Student-Newman-Keuls test. Correlations between ARFI and conventional US parameters with variables (glomerular sclerosis, tubulointerstitial damage, vascular sclerosis, histologic scores, serum creatinine, and e-GFR) were analyzed by using Pearson's correlation coefficients.

The diagnostic performance of ARFI imaging and conventional US in identifying renal histological fibrosis was assessed by receiver operating characteristic (ROC) curves. The optimal cut-off values for the prediction of different group with CKD were chosen to maximize the sum of sensitivity and specificity. Sensitivity, specificity, and positive and negative predictive values were calculated after the cut-off values were optimized. *P*<0.05 was considered statistically significant.

Intraclass correlation coefficients (ICCs) were used to assess the intra- and inter-observer reliability of the SWV measurements. Agreement was indicative of poor (ICC<0.40), fair to good (ICC = 0.40 to 0.75) or excellent (ICC>0.75).

## Results

### Patient characteristics

Patients' demographic, pathological, biochemical, ARFI and conventional US characteristics are shown in Tables 2 and 3. There was no significant difference in gender, age, and SWV measurement depth among these groups. According to the histologic scores, the 163 patients were classified as follows: 97 patients were classified as mildly impaired, 38 as moderately impaired, and 28 as severely impaired group.

**Table 2:** Characteristics of the participants.

Characteristic	Controls (n = 32)	Patient groups (n = 163)		
		Mildly impaired	Moderately impaired	Severely impaired
		(n = 97)	(n = 38)	(n = 28)
Female/male	17/15	42/55	16/22	14/14
Age, year	41.53±13.12*	38.81±15.62	42.50±15.78	48.14±16.67
Serum creatinine, umol/L	60.16±9.36	80.45±31.98	183.92±230.76^b,d^	591.96±409.78^c,e,f^
e-GFR, ml/min/1.73 m^2^	120.39±24.98	83.17±25.89^ c^	48.23±24.41^c,e^	13.06±9.62^c,e,f^
SWV, m/s	2.81±0.36	2.60±0.37^ a^	2.47±0.39^ c^	2.00±0.29^c,e,f^
SWV-depth, cm	4.18±1.14	3.79±0.97	4.03±0.3	4.08±0.93
Renal length, mm	101.03±6.65	105.16±9.28	103.45±10.31	89.75±16.44^c,e,f^
Parenchymal thickness, mm	15.75±1.67	15.94±2.34	15.68±2.49	12.75±3.74^c,e,f^
Interlobar arterial RI	0.59±0.49	0.60±0.06	0.62±0.07	0.70±0.09^c,e,f^

^*^ Variables are expressed as Mean ± SD.

Compared with controls: ^a^ *P*<0.05, ^b^ *P*<0.01, ^c^ *P*<0.001; compared with mildly impaired group: ^d^ *P*<0.01, ^e^ *P*<0.001; compared with moderately impaired group: ^f^ *P*<0.001.

**Table 3:** Pathologic diagnosis of CKD patients.

Diagnosis	Number
Membranous glomerulonephritis	54
IgA nephropathy	25
Minimal change nephrosis	13
Focal segmental glomerulosclerosis	19
Lupus nephritis	22
Crescentic glomerulonephritis	1
Diabetic nephropathy	13
Interstitial disease	5
Mesangial proliferative glomerulonephritis	8
Nephrosclerosis	2
Purpura nephritis	1

### Relationship of SWV measurements with pathological parameters and laboratory tests

**Figure 1:**
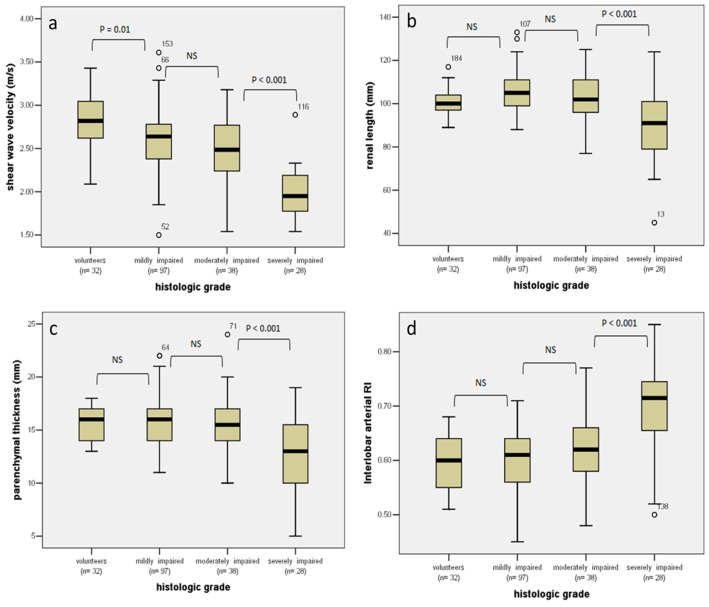
Box plots of SWV (a), renal length (b), parenchymal thickness (c), and interlobar arterial RI (d) in different groups. Top and bottom of the boxes represent the first and third quartiles. The thick line through each box marks the median value. Error bars represent the minimum and maximum values (range). Small circles show outliers. The numbers above the circles represent the No of the data. NS  =  Non-significant.

**Figure 2:**
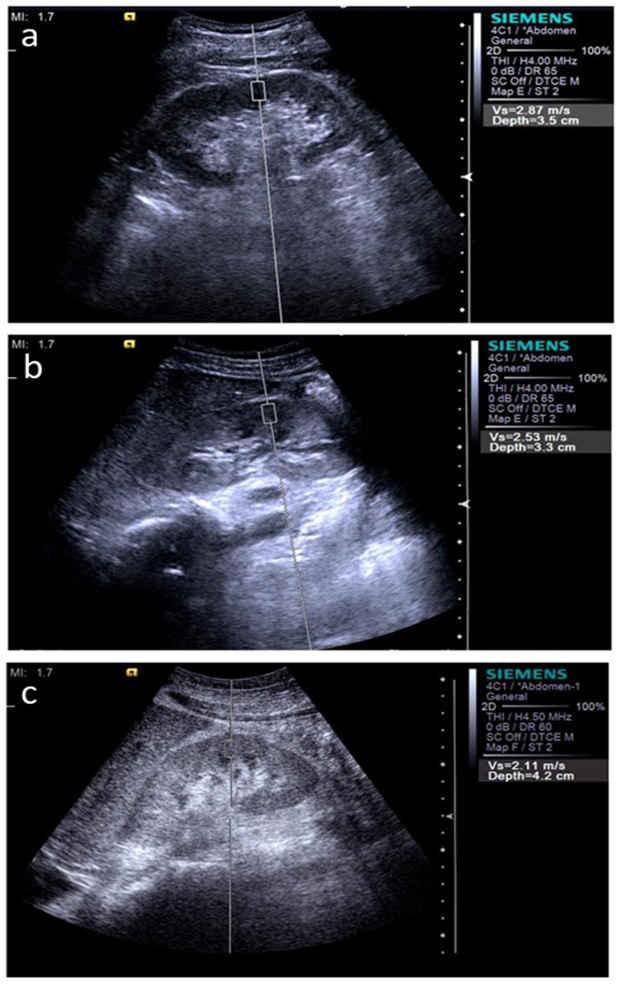
ARFI images in the mildly (a), moderately (b), and severely (c) impaired kidney. The SWV values were 2.87 m/s, 2.53 m/s, and 2.11 m/s, respectively.

In CKD patients, SWV measurements correlated significantly with glomerular sclerosis (*r* = −0.492, *P*<0.001), tubulointerstitial damage (*r* = −0.501, *P*<0.001), vascular sclerosis (*r* = −0.422, *P*<0.001), histologic scores (*r* = −0.511, *P*<0.001), serum creatinine (*r* = −0.503, *P*<0.001), and e-GFR (*r* = 0.587, *P*<0.001) (Table 4). The mean values (95% CI) of SWV measurements in the 32 healthy volunteers was 2.81 m/s (2.68–2.94 m/s), and in kidneys with mildly, moderately, and severely impaired were 2.60 m/s (2.53–2.67 m/s), 2.47 m/s (2.35–2.60 m/s), and 2.00 m/s (1.89–2.11 m/s), respectively (Fig. 1). One-way ANOVA indicated significant differences between the controls with different group in CKD patients (all *P*≤0.01). The mean SWV in kidneys with severely impaired was significant lower than that mildly or moderately impaired group (both *P*<0.001) (Fig. 2). However, there was no statistical difference observed between mildly and moderately impaired kidneys (*P* = 0.072).

**Table 4:** Correlation of ARFI and conventional US parameters with different variables in the 163 CKD patients.

	Pearson correlation coefficients					
	Glomerular sclerosis	Tubulointerstitial damage	Vascular sclerosis	Histologic scores	Serum creatinine	e-GFR
SWV	−0.492	−0.501	−0.422	−0.511	−0.503	0.587
	(*P*<0.001)	(*P*<0.001)	(*P*<0.001)	(*P*<0.001)	(*P*<0.001)	(*P*<0.001)
Renal length	−0.361	−0.419	−0.396	−0.416	−0.400	0.370
	(*P*<0.001)	(*P*<0.001)	(*P*<0.001)	(*P*<0.001)	(*P*<0.001)	(*P*<0.001)
Parenchymal thickness	−0.330	−0.349	−0.363	−0.368	−0.394	0.297
	(*P*<0.001)	(*P*<0.001)	(*P*<0.001)	(*P*<0.001)	(*P*<0.001)	(*P*<0.001)
Interlobar arterial RI	0.473	0.467	0.333	0.468	0.459	−0.436
	(*P*<0.001)	(*P*<0.001)	(*P*<0.001)	(*P*<0.001)	(*P*<0.001)	(*P*<0.001)

### Relationship of Conventional US with pathological parameters and laboratory tests

Table 4 shows the correlations of renal length, parenchymal thickness, and interlobar arterial RI with each pathological parameter, serum creatinine, and e-GFR. In comparison with the conventional parameters, ARFI imaging showed a better correlation with pathological parameters and the laboratory tests. The renal length and parenchymal thickness were significantly reduced in the severely impaired kidneys, compared with that of mildly, moderately impaired kidneys and health volunteers (all *P*<0.001). RI of interlobar arterial in severely impaired kidneys was significantly higher than the other patient groups and controls (all *P*<0.001). However, no significant difference was found in these conventional US parameters among mildly, moderately impaired groups, and controls (Fig. 1).

### Comparison of ARFI with conventional US

**Figure 3:**
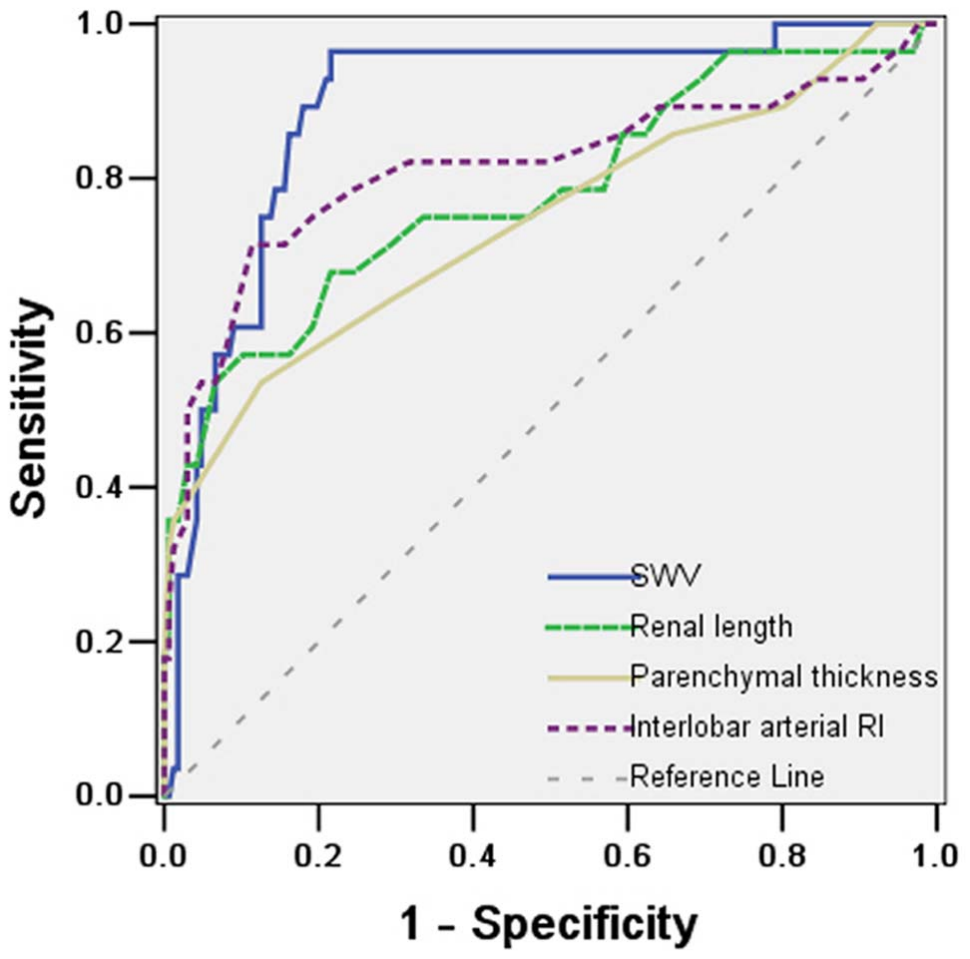
Receiver operating characteristic curves for SWV, renal length, parenchymal thickness, and interlobar arterial RI in the diagnosis of severely impaired kidney (histologic score ≥ a19 points).

The areas under the ROC curves, by ARFI imaging, renal length, parenchymal thickness, and interlobar arterial RI were 0.735, 0.430, 0.553, and 0.601, respectively, for the diagnosis of mildly impaired kidneys; 0.744, 0.630, 0.625, and 0.684, for the diagnosis of moderately impaired kidneys; and 0.895, 0.781, 0.744, and 0.814, respectively, for the diagnosis of severely impaired kidneys. The area under the ROC curve of SWV was superior to those stratified by conventional US parameters (Table 5, Fig. 3).

**Table 5:** Diagnostic Accuracy of SWV, Renal length, Parenchymal thickness, and Interlobar arterial RI in different histolotical fibrosis grades.

	Mildly impaired		Moderately impaired		Severely impaired	
	AUC	*P*	AUC	*P*	AUC	*P*
SWV	0.735 (0.644–0.826)	<0.001	0.744 (0.668–0.819)	<0.001	0.895 (0.833–0.956)	<0.001
Renal length	0.430 (0.340–0.520)	0.211	0.630 (0.542–0.719)	0.003	0.781 (0.674–0.888)	<0.001
Parenchymal thickness	0.553 (0.453–0.652)	0.354	0.625 (0.539–0.710)	0.004	0.744 (0.629–0.859)	<0.001
Interlobar arterial RI	0.601 (0.506–0.696)	0.072	0.684 (0.599–0.770)	<0.001	0.814 (0.704–0.924)	<0.001

Note. - Numbers in parentheses are 95% confidence intervals.

AUC, area under receiver operating characteristic curve.

When choosing to maximize the sum of sensitivity and specificity, the optimal cut-off values of ARFI imaging were determined to be 2.65 m/s for mildly impaired kidneys, 2.50 m/s for moderately impaired kidneys, and 2.33 m/s for severely impaired kidneys, respectively. The corresponding sensitivity, specificity, and positive and negative predictive values were cited in Table 6.

**Table 6:** ARFI imaging for the diagnosis of renal histological fibrosis.

	Mildly impaired	Moderately impaired	Severely impaired
Cut-off value (m/s)	2.65	2.50	2.33
Sensitivity (%)	63.8	71.2	96.4
Specificity (%)	75.0	69.8	78.4
Positive predictive value (%)	92.9	57.7	42.9
Negative predictive value (%)	28.9	82.6	99.2

### Reproducibility with SWV measurements

The correlation coefficients were 0.709 (95% CI: 0.390–0.859, *P*<0.001) for intra-observer reliability and 0.627 (95% CI: 0.233–0.818, *P* = 0.004) for inter-observer reliability, respectively. This indicated the SWV measurements had fair to good reproducibility.

## Discussion

Acoustic radiation force impulse imaging is a newly developed, noninvasive technique to quantitatively assess the mechanical stiffness properties of tissues. It uses short-duration (approximately 0.3 s), high-intensity pushing pulses of acoustic radiation force to generate localized displacements in tissue and conventional US beams to track the tissue dynamic response. The stiffer the tissue is, the faster is the SWV [9], [21]. Since the radiation force of acoustic pulses induces the mechanical vibrations automatically, ARFI is operator-independent and can determine tissue elasticity quantitatively [22]. It overcomes the intrinsic limitations of previously strain elastography, which can only determine qualitative and relative elasticity. Moreover, ARFI imaging uses a conventional convex or linear array probe, allowing its incorporation into routine sonographic examinations [23].

In the field of nephrology, several studies have conducted to apply ARFI imaging for noninvasive evaluation of renal allografts fibrosis in the transplanted kidneys. Nevertheless, these studies have yielded conflicting results. He et al. [24] indicated that at a cut-off value of 2.625 m/s in diagnosing renal allograft dysfunction, the sensitivity and specificity were 72.0% and 86.5%, respectively, much better than the values for RI, which sensitivity and specificity were 62.0% and 69.2% by using a cut-off value of 0.625. Syversveen et al. [15] reported that SWV did not differ significantly in transplant kidneys with and without fibrosis. The value of ARFI in noninvasive assessment of renal fibrosis has rarely been studied in CKD patients and remains ill-defined. In the literature, only two studies investigated the correlation of SWV value with histological fibrosis idicators in patients with CKD [16], [25]. Their sample size was relatively small. The optimal cut-off values for different grades of renal fibrosis have not been defined yet.

In the present study, we first investigate the accuracy of ARFI elastography, compared with conventional US, in the assessment of renal histological fibrosis in CKD patients. Results revealed that the mean SWV of the renal parenchyma had statistical difference between the health volunteers with different group in CKD patients (all *P*≤0.01). SWV values correlate significantly with pathological parameters (*r* = −0.422–−0.511, *P*<0.001), serum creatinine (*r* = −0.503, *P*<0.001), and glomerular filtration rate (*r* = 0.587, *P*<0.001). Similar result was demonstrated in Guo et al.'s evaluation that the SWV correlate significantly with e-GFR (*r* = 0.3, *P* = 0.018), serum urea nitrogen (*r* = −0.3, *P* = 0.016) and creatinine (*r* = −0.41, *P* = 0.001) [14]. In contrast to our findings, however, Wang et al. [16] recently described that ARFI values did not correlate with any pathological indicators of fibrosis in CKD patients, although a standardized measurement protocol was employed. One explanation for these inconsistent results may be the relatively small number of patients in Wang et al.'s study. Another hypothesis was that the structural heterogeneity of renal parenchyma and the diminished renal blood flow impacted on the SWV values in patients with CKD [26].

In ARFI elastography for chronic liver diseases, SWV showed a good positive correlation with the stage of hepatic fibrosis [27]. Kircheis et al. [28] indicated that liver stiffness values increased significantly as fibrosis progressed. Mean SWV was 1.09±0.13 m/s for patients with no significant fibrosis, 1.44±0.26 m/s for patients with significant liver fibrosis, and 2.55±0.77 m/s for patients with liver cirrhosis, respectively. In contrast to those findings for chronic liver diseases, the SWV in CKD patients was significantly lower than that in healthy controls in our study. A negative correlation was found between the SWV and renal fibrosis of CKD. The reason for this difference remains unclear. Some scholar speculated the differences of histological changes and mechanical property between kidney and liver result in this inconsistency [14]. Asano et al. [26] reported the degree of interstitial fibrosis in the kidneys of CKD is not as marked as that in chronic liver disease and suspected interstitial fibrosis is not the main affecting factor of ARFI elastography in the kidneys tissue elasticity.

Conventional US can provide effective information on renal size, parenchyma thickness, and the vascular status of the kidney, which are quite helpful in the detecting irreversible parenchymal damage of renal failure. In a study by Moghazi et al. [6], renal size and parenchymal thickness were significantly correlated with glomerular sclerosis and tubular atrophy. Sugiura et al. [7] stated that an increased RI of the interlobar arteries was correlated with glomerulosclerosis, tubulointerstitial damage, and vascular lesions. Unfortunately, all these parameters are still limited by a lack of sensitivity and specificity and cannot be objectively quantified according to a universally accepted standard [14], [24]. Compared to ARFI imaging, our findings confirmed that the renal length, parenchymal thickness, and interlobar arterial RI showed statistically, but weaker correlation with pathological parameters, serum creatinine, and e-GFR. ROC analyses indicated that the area under the ROC curve for the diagnosis of renal histological fibrosis using ARFI imaging was superior to these conventional US parameters.

Although the potential of ARFI elastography is encouraging, the shortcomings of this new technique should be taken into consideration. such as the area of the ROI is fixed (1 cm×0.6 cm), detected depth is limit to 8 cm, and wide intra- and inter-observer variation in assessment of renal stiffness. Some scholar indicated that SWV measurements in kidney transplants are dependent on the applied transducer force [29]. In the animal experimental research, Gennisson et al. [30] demonstrated renal elasticity is directly proportional to the urinary and vascular pressure. Elasticity values were always higher when acquisitions were achieved with the ultrasonic main axis perpendicular to main pyramid axis. Furthermore, kidneys highly anisotropy, due to the complex architecture composed of glomeruli, tubuli, blood vessels, and stromal components, could be an important factor resulting in the wide intra- and inter-observer variation [31]. Even though these defects are still impossible to completely eliminate, in our present study, we repeated SWV measurements by the same operator to eliminate inter-observer variation of measurements. ROI was positioned in the outer renal cortex and carefully avoided the renal medulla and sinus to reduce the physiological heterogeneity of the tissue. And then, an acceptable intra- and inter- observer agreement was achieved, with intraclass coefficient correlation were 0.709 (95% CI: 0.390–0.859, *P*<0.001) and 0.627 (95% CI: 0.233–0.818, *P* = 0.004), respectively.

There are some limitations to our study. First, there was a certain degree of overlap between SWVs in different grades of fibrosis. It remains to be determined whether ARFI imaging is sufficiently effective to avoid renal biopsy in clinical practice. Second, it lacks evaluation of pathological types in CKD patients. Nevertheless, previous study has reported that different pathological types of CKD share similar pathogenic features, such as glomerulosclerosis, tubular atrophy, and interstitial fibrosis [15]. There is a possibility that the SWV values would be decreased similarly regardless of the pathological types. Further study could be performed to confirm this hypothesis. Finally, the potential influencing factors (ie, age, gender, BMI, and systolic blood pressure) on SWV measurements need further investigation in CKD patients.

In conclusion, in the present study, ARFI quantification was superior to conventional US in the assessment of renal histological fibrosis. Despite its limitations at present stage, ARFI may be an effective tool for non-invasive evaluating and classifying histological fibrosis of CKD.
